# Association of Obstructive Sleep Apnea-Hypopnea Syndrome with hearing loss: A systematic review and meta-analysis

**DOI:** 10.3389/fneur.2022.1017982

**Published:** 2022-10-19

**Authors:** Chaoyu Wang, Fu Xu, Mingdi Chen, Xiaojuan Chen, Chunhe Li, Xishi Sun, Yu Zhang, Huizhao Liao, Qinglan Wu, Huimin Chen, Shunhong Li, Jinru Zhu, Junyan Lin, Xudong Ou, Zhihong Zou, Yuming Li, Riken Chen, Zhenzhen Zheng, Yang Wang

**Affiliations:** ^1^The Second Affiliated Hospital of Guangdong Medical University, Zhanjiang, China; ^2^Department of Respiratory and Critical Care Medicine, Taishan Hospital of Traditional Chinese Medicine, Jiangmen, China; ^3^Department of Otolaryngology-Head and Neck Surgery, The Affiliated Shunde Hospital of Jinan University, Foshan, China; ^4^Medical College, Jiaying University, Meizhou, China; ^5^The First Affiliated Hospital of Guangzhou University of Chinese Medicine, Guangzhou, China; ^6^Department of Emergency, Affiliated Hospital of Guangdong Medical University, Zhanjiang, China; ^7^State Key Laboratory of Respiratory Disease, National Clinical Research Center for Respiratory Disease, Guangzhou Institute of Respiratory Health, The First Affiliated Hospital of Guangzhou Medical University, Guangzhou Medical University, Guangzhou, China; ^8^Department of Respiratory and Critical Care Medicine, Central People's Hospital of Zhanjiang, Zhanjiang, China; ^9^Department of Ophthalmology, Xinhui Chinese Traditional Hospital, Jiangmen, China; ^10^Department of Respiratory and Critical Care Medicine, The First People's Hospital of Chongqing Liangjiang New Area, Chongqing, China

**Keywords:** Obstructive Sleep Apnea-Hypopnea Syndrome, meta-analysis, hearing loss (HL), incidence, sleep

## Abstract

**Objective:**

This study seeks to investigate the relationship between Obstructive Sleep Apnea-Hypopnea Syndrome (OSAHS) and hearing impairment by meta-analysis.

**Methods:**

Cochrane Library, PubMed, Embase, Web of Science and other databases are searched from their establishment to July 1st, 2022. Literature on the relationship between OSAHS and hearing loss is collected, and two researchers independently perform screening, data extraction and quality evaluation on the included literature. Meta-analysis is performed using RevMan 5.4.1 software. According to the heterogeneity between studies, a random-effects model or fixed-effects model is used for meta-analysis.

**Results:**

A total of 10 articles are included, with 7,867 subjects, 1,832 in the OSAHS group and 6,035 in the control group. The meta-analysis shows that the incidence of hearing impairment in the OSAHS group is higher than in the control group (OR = 1.38; 95% CI 1.18–1.62, Z = 4.09, *P* < 0.001), and the average hearing threshold of OSAHS patients is higher than that of the control group (MD = 5.89; 95% CI 1.87–9.91, Z = 2.87, *P* = 0.004). After stratifying the included studies according to hearing frequency, the meta-analysis shows that the OSAHS group has a higher threshold of 0.25, and the response amplitudes at frequencies 2, 4, 6, and 8 kHz are all higher than those of the control group.

**Conclusion:**

Compared with the control group, the OSAHS group has a higher incidence of hearing loss, mainly high-frequency hearing loss. Thus, OSAHS is closely associated with and a risk factor for hearing loss.

## Introduction

Obstructive Sleep Apnea-Hypopnea Syndrome (OSAHS) refers to either hypoventilation (reduced airflow during sleep) or apnea (complete cessation of airflow during sleep) due to the repeated collapse and obstruction of the upper airway (1, 2). Its clinical manifestations include snoring, frequent awakening and morning sleepiness. OSAHS is currently considered a cause of multisystem disease (3) as it can cause cardiovascular disease, stroke, daytime sleepiness, cognitive dysfunction and immune and endocrine system imbalances. It also involves many disciplines such as ophthalmology, stomatology and ENT. Hearing loss, as one of the common manifestations of ENT diseases, is pathological decreased hearing sensitivity of the human ear, including mild to severe hearing loss and tinnitus. Studies have shown that hearing loss not only affects people's cognitive and social communication ability (4–6), but also their mental health and social adaptability (7–9), leading to many problems in job and career development, including employment difficulties, low income and job status, and reduced opportunities for training and re-education (10). On March 3rd, 2021, WHO released the world's first report on hearing (11). According to the report, more than 1.5 billion people in the world are suffering from hearing loss to varying degrees, of which 430 million have moderate to severe hearing loss. This number could increase to 2.5 billion by 2050.

Several studies have explored the relationship between OSAHS and hearing loss, and the understanding of the relationship between the two is relatively late. A search of the PubMed database found that most studies on this topic were published after 2003. The conduction mechanism of the inner ear and the transmission of nerve impulses are highly dependent on the supply of oxygen. The conduction mechanism of the inner ear and the transmission of nerve impulses along the hearing are highly dependent on the supply of oxygen, so any significant reduction in local oxygen may have a significant effect on hearing sensitivity. The possibility that OSAHS interferes with the generation and transmission of nerve impulses at the level of the auditory system is currently under consideration (12–14). Kayabasi et al. (15) found that OSAHS has multiple effects on hearing, and hearing impairment may be related to the severity of OSAHS. Moderate OSAHS affects high-frequency hearing function, and severe OSAHS negatively affects all hearing functions. Peripheral auditory pathways and brainstem assessments were studied in OSAHS patients by setting an observer with OSAHS and a control group without OSAHS, Matsumura et al. (16) found that there was no difference between the groups for hearing thresholds, tympanometry and evaluated Brainstem Auditory Evoked Response parameters. Despite the association between OSAHS and a variety of comorbidities, there are limited studies evaluating the relationship between OSAHS and hearing with varying results, and there is not an adequate literature review on this topic. The results of these studies are inconsistent, which not only caused great distress to clinicians, but also affected the decision-making of hearing loss in patients with OSAHS.

The primary purpose of this systematic review was to explore the OSAHS-based alteration in the auditory system function by conducting a quantitative analysis of presently published data. We compared PTA thresholds in OSAHS patients and controls. Besides, the results of this review may provide an insight into the potential association between OSAHS and hearing loss, thereby providing evidence-based medical evidence for early prevention of hearing loss in patients with OSAHS.

## Materials and methods

### Search strategy

According to the Meta-analysis of Observational Studies in Epidemiology (MOOSE) statement (17) and Standard of Preferred Reporting Items for Systematic Reviews and Meta-Analyses (PRISMA) (18), the Cochrane Library, PubMed, Embase and Web of Science databases were searched from their establishment to July 1st, 2022. English search terms included “Sleep Apnea, Obstructive”; “OSA”; “OSAH”; “OSAHS”; “Apneas, Obstructive Sleep”; “Obstructive Sleep Apneas”; “Sleep Apneas, Obstructive”; “Obstructive Sleep Apnea Syndrome”; “Hearing Loss”; “Loss, Hearing”; “Hypoacusis”; “Hypoacuses”; “Hearing Impairment”; and “Transitory Deafness.” In order to avoid missing literature and missing data, the references to included studies were searched again. The type of research design was not limited, the language of the literature was English and the research objects were humans. The protocol was registered in the Prospective Register of Systematic Reviews (ProsperoCRD42022344836).

### Inclusion and exclusion criteria

Inclusion criteria: ① the subjects were adults over 18 years old; ② the subjects were OSAHS patients clinically diagnosed in the case group, and those without OSAHS in the control group; ③ the study types were cohort study, case-control study and cross-sectional study, which were compared with the control group; ④ the exposure factors were the number of patients with hearing loss and/or pure-tone audiometry indicators.

Exclusion criteria: ① repeated published and included studies; ② studies without a control group; ③ reviews, conference abstracts or literature for which the full text could not be obtained; ④ studies with incomplete data or data that could not be extracted wherein the original authors were contacted and did not respond or could not provide meta-analysis data; ⑤ studies with little research information, incomplete data or inconsistent outcome indicators; ⑥ studies in which the subjects had other diseases that could affect hearing function, such as outer or middle ear diseases, high environmental noise exposure or ototoxic drug exposure; ⑦ studies in languages other than English.

### Literature screening

The data was screened and extracted from all eligible studies by two researchers, then checked against each other. Differences in extracted data were discussed among authors, and studies with differences were provided to a third researcher for analysis to decide whether they should be included. The methodological quality of the included literature was assessed.

### Evaluation of literature quality

The Newcastle-Ottawa scale (NOS) was used to evaluate the quality of the included cohort studies and cross-sectional studies (19). NOS assigns one ⋆ for each criterion that a study meets. If a criterion is not met, one ⋆ is assigned. After the evaluation, the more stars (⋆), the higher the quality. The highest score is 9 ⋆, and it is generally considered that 5 ⋆ and above indicates that the research quality is medium to high. The quality evaluation was conducted independently by two researchers at the same time, and disagreements were resolved through discussion with a third researcher. Studies with a score of 7 or higher were considered high quality.

### Literature data extraction

Data extracted from the literature included the first author, study area, publication time, sample size, the total number of cases and mean and standard deviation of study effect indicators. After data extraction, the two datasets were checked and inconsistent data was extracted again. After checking, the data was analyzed.

### Ending and exposure

OSAHS was assessed by polysomnography (PSG) or sleep questionnaire, and hearing loss was assessed by pure-tone audiometry. In the included studies, the total number of cases of hearing loss and mean and standard deviation of pure-tone hearing thresholds in the OSAHS and control groups were counted.

### Statistical methods

Statistical analysis was performed using R 5.4.1 software. Sensitivity analysis and publication bias detection were performed using Stata 12.0 software. The heterogeneity of the studies was analyzed by the *I*^2^ statistical value test and Q test. If the heterogeneity was not statistically significant (*I*^2^ ≤ 50% and *P* ≥ 0.1), a fixed-effects model was used; if there was heterogeneity (*I*^2^ > 50% and *P* < 0.1), a random-effects model was used (20). When heterogeneity occurred, subgroup analysis and sensitivity analysis were performed to find the source. Publication bias was detected using Egger's method. Enumeration data was analyzed using odds ratio (OR) and 95% confidence interval (CI). Data measurement was carried out by weighted mean difference (MD) and 95% CI, and *P* < 0.05 was considered statistically significant.

## Results

### Literature screening results

**Figure 1:**
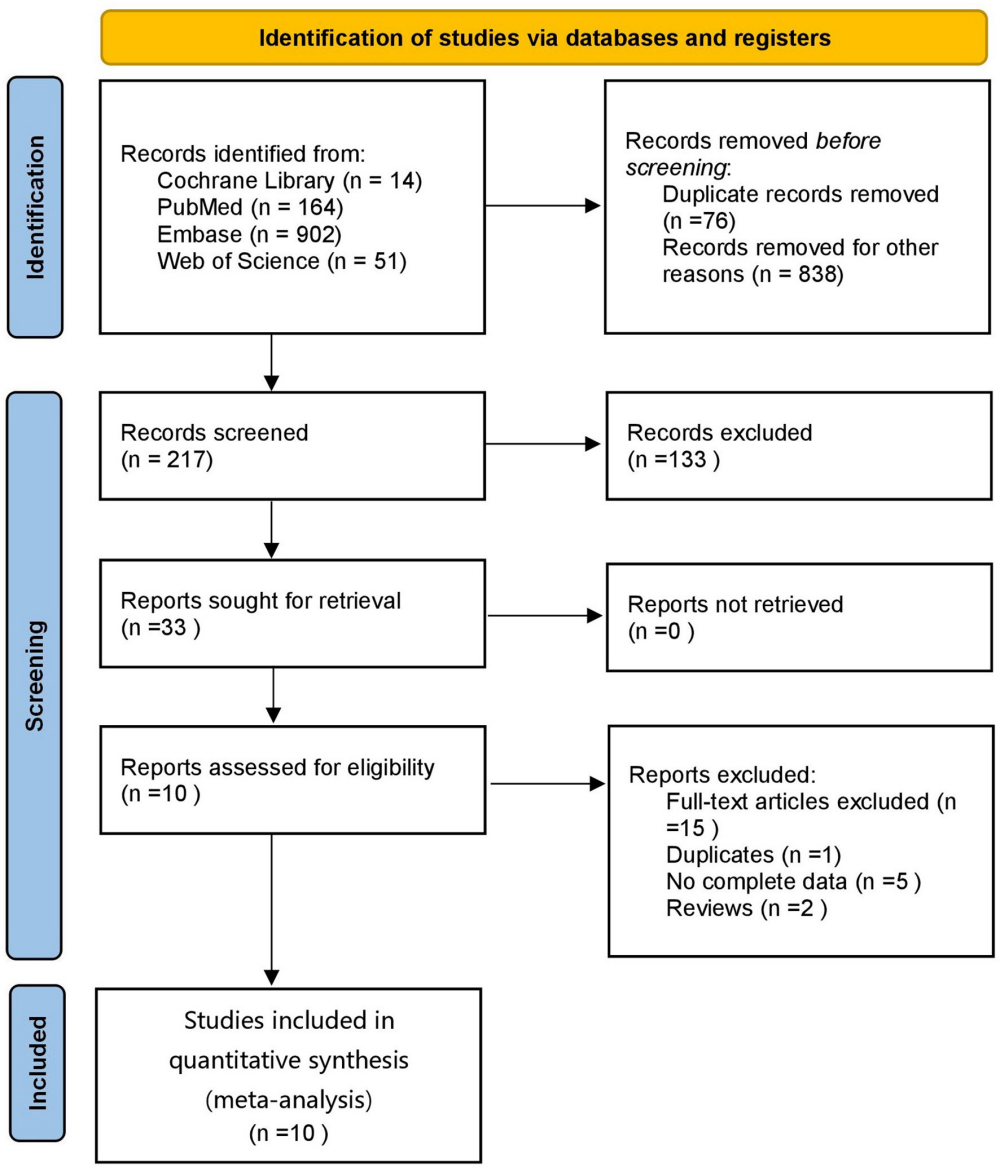
Flow diagram of literature screening.

A total of 1,131 articles were retrieved, of which 1,055 were obtained after deduplication, 838 were removed by reading the titles and abstracts, and ten were finally included after reading the full texts (21–30) (Figure 1). A total of 7,867 subjects were included. The study populations were from China, Taiwan (China), Italy, Spain, France, Turkey and the Czech Republic. The basic characteristics of the literature included in the study are shown in Table 1.

**Table 1:** Basic features of the included studies.

**References**	**Country**	**Sample size**	**Age**		**Sex (M/F)**		**OSAHS**	**Outcome**
	**region**	**(case group/**					**assessment**
		**control group)**					
			**Case**	**Control**	**Case**	**Control**		
			**group**	**group**	**group**	**group**		
Gallina et al. (21)	Italy	45/30	43.3 (24–56)	41 (27–54)	31/24	–	Sleep monitoring	
Hwang et al. (22)	Taiwan, China	34/190	62.2 ± 5.5	61.1 ± 6.9	28/6	98/92	PSG	
Casale et al. (23)	Spain	18/21	31	39	15/3	16/5	PSG	
Martines et al. (24)	Italy	100/60	46.19 ± 7.01*		103/57*		PSG	
Deniz et al. (25)	Turkey	120/40	42.4 (23–55)*		84/76*		PSG	
Ekin et al. (26)	Turkey	27/39	41.56 ± 8.99	39.14 ± 9.91	–	–	PSG	
Vorlova et al. (27)	Czech Republic	28/15	(AHI 5–15)46.4 ± 9.2	47.3 ± 8.0	43/0*		PSG	
			(AHI ≥ 30)50.8 ± 10.2	
Lisan et al. (28)	France	1,267/5,530	60.1 ± 6.2	59.4 ± 6.2	942/325	3,375/2,155	Berlin questionnaire	
Li et al. (29)	China	58/20	40.4 ± 11.3	–	46/6	–	PSG	
Gozeler and Sengoz (30)	Turkey	35/30	44.4 ± 3.9	43.1 ± 2.4	20/15	13/17	PSG	

^*^Total study population data (including case and control groups).

PSG, Polysomnography; ① Incidence of hearing loss; ② Average hearing threshold.

### Quality evaluation of included studies

The NOS scale was used to evaluate the quality of the included observational studies. The specific evaluation is shown in Table 2. The lowest overall evaluation was 7⋆ and the highest was 9⋆, all of which were of high quality. All studies had a low to moderate risk of bias and no studies were excluded due to poor quality (< 5⋆).

**Table 2:** Newcastle–Ottawa scale of the included studies.

**References**	**Selection**	**Comparability**	**Exposure**	**Quality**
				**scores**
Gallina et al. (21)	⋆⋆⋆⋆	⋆	⋆⋆	7
Hwang et al. (22)	⋆⋆⋆	⋆	⋆⋆⋆	7
Casale et al. (23)	⋆⋆⋆⋆		⋆⋆⋆	7
Martines et al. (24)	⋆⋆⋆⋆		⋆⋆⋆	7
Deniz et al. (25)	⋆⋆⋆⋆	⋆	⋆⋆⋆	8
Ekin et al. (26)	⋆⋆⋆⋆	⋆⋆	⋆⋆⋆	9
Vorlova et al. (27)	⋆⋆⋆	⋆⋆	⋆⋆⋆	8
Lisan et al. (28)	⋆⋆⋆⋆	⋆	⋆⋆⋆	8
Li et al. (29)	⋆⋆⋆⋆		⋆⋆⋆	7
Gozeler and Sengoz (30)	⋆⋆⋆	⋆	⋆⋆⋆	7

### Statistical analysis results

#### Correlation between OSAHS and the incidence of hearing loss

**Figure 2:**
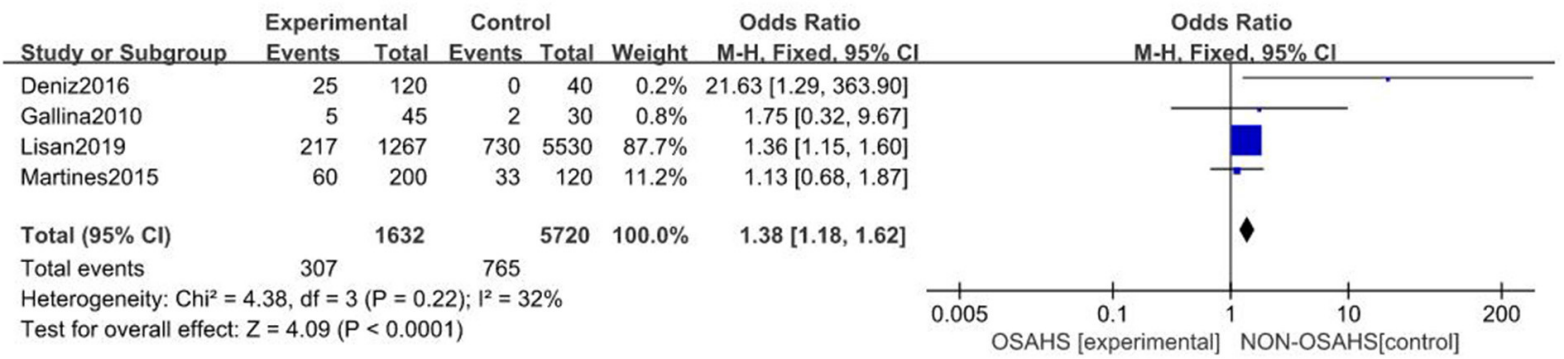
Forest plot of correlation between the incidence of hearing loss in OSAHS group and control group.

A total of four studies were included (21, 24, 25, 28) with 7,352 subjects (Figure 2). There was slight heterogeneity among the included studies (*P* = 0.22, *I*^2^ = 32%), so a fixed-effects model was used for analysis. The results showed that the incidence of hearing loss in the OSAHS group was higher than that in the control group (OR = 1.38; 95% CI 1.18–1.62, Z = 4.09, *P* < 0.001). Thus, OSAHS is associated with and a risk factor for hearing loss.

#### Correlation between OSAHS and pure-tone hearing threshold

**Figure 3:**
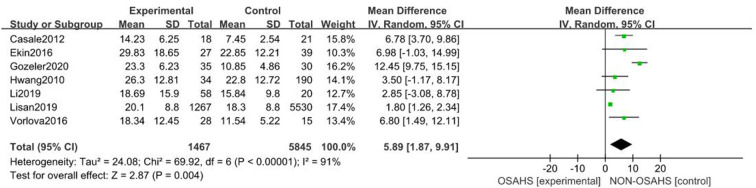
Forest plot of pure-tone hearing threshold correlation between OSAHS group and control group.

**Figure 4:**
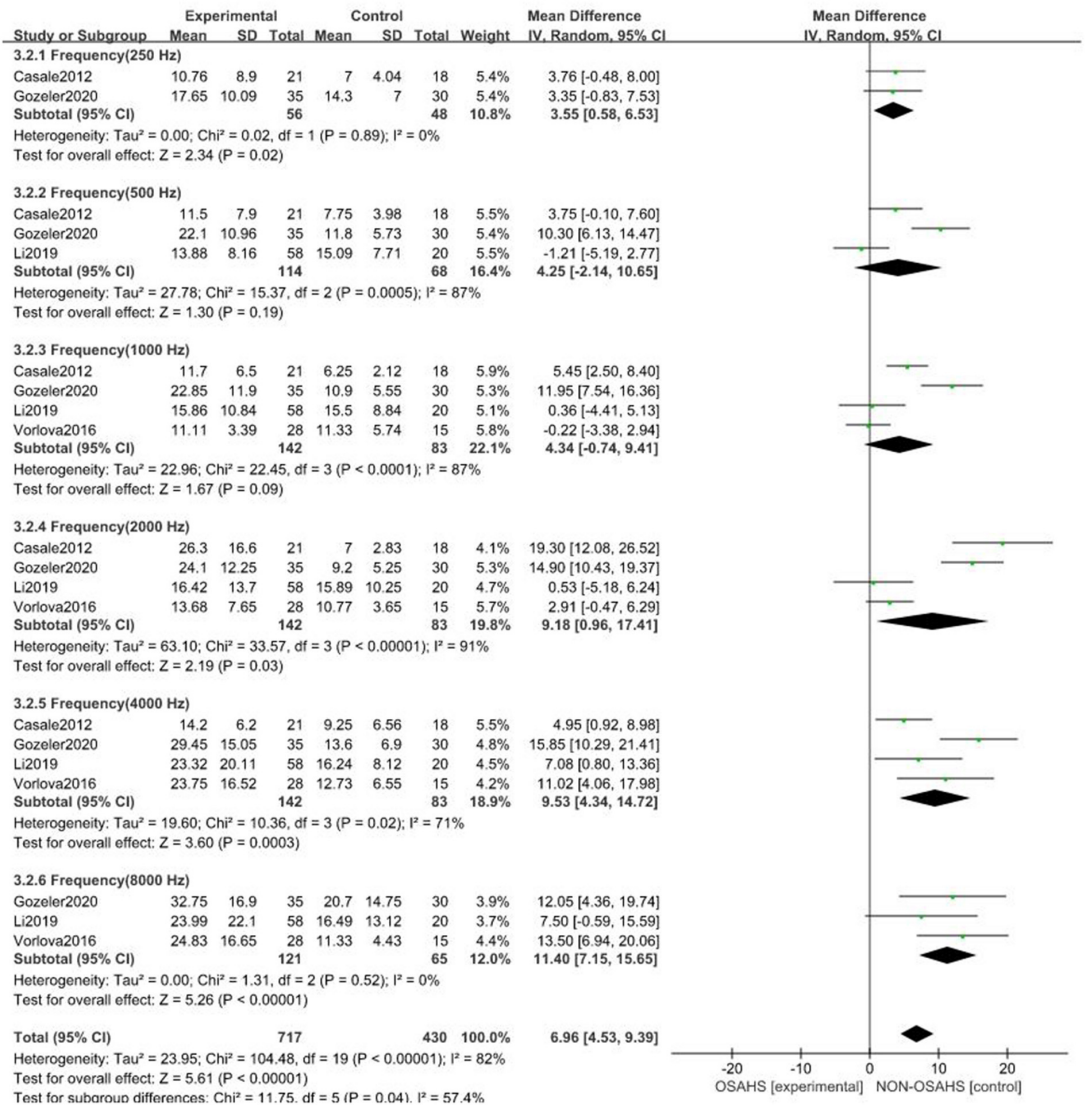
Forest plot of pure-tone hearing threshold correlation between OSAHS group and control group with different audiometric test frequencies.

A total of seven studies were included (22, 23, 26–30) with 7,312 subjects (Figure 3). There was great heterogeneity among the included studies (*P* < 0.001, *I*^2^ = 91%), so a random-effects model was used for analysis. The results showed that compared with the control group, the OSAHS group had a significantly higher pure-tone hearing threshold (MD = 5.89; 95% CI 1.87–9.91, Z = 2.87, *P* = 0.004) with statistical significance. To further reduce clinical heterogeneity and explore the effects of OSAHS on pure-tone hearing thresholds at different audiometric test frequencies, a subgroup analysis of pure-tone hearing thresholds with different hearing test frequencies was conducted. A total of four studies reported pure-tone hearing threshold data of different hearing test frequencies between the OSAHS group and the control group. The total heterogeneity was (*P* < 0.001, *I*^2^ = 82%), so a random-effects model was used for analysis (Figure 4). There were two studies (21, 28) on the 250 Hz hearing test with a sample size of 104 cases. The results showed no heterogeneity between studies (*P* = 0.89, *I*^2^ = 0%), and the pure-tone hearing threshold of the OSAHS group was higher than that of the control group (MD = 3.55; 95% CI 0.58–6.53, Z = 2.34, *P* = 0.02).

There were three studies on the 500 Hz hearing test (23, 29, 30) with a sample size of 182 cases. There was heterogeneity between studies (*P* < 0.001, *I*^2^ = 87%). The results showed that the pure-tone hearing threshold of the OSAHS group was higher than that of the control group (MD = 4.25; 95% CI −2.14 to 10.65, Z = 1.3, *P* = 0.19), but the results were not statistically significant.

There were four studies on the 1,000 Hz hearing test (23, 27, 29, 30) with a sample size of 225 cases. There was large heterogeneity between studies (*P* < 0.001, *I*^2^ = 87%). The results showed that the pure-tone hearing threshold of the OSAHS group was higher than that of the control group (MD = 1.67; 95% CI −0.74 to 9.41, Z = 1.67, *P* = 0.09), but the results were not statistically significant.

There were four studies on the 2,000 Hz hearing test (23, 27, 29, 30) with a sample size of 225 cases. There was large heterogeneity between studies (*P* < 0.001, *I*^2^ = 91%). The results showed that the pure-tone hearing threshold of the OSAHS group was higher than that of the control group (MD = 9.18; 95% CI 0.96–17.41, Z = 2.19, *P* = 0.03).

There were four studies on the 4,000 Hz hearing test (23, 27, 29, 30) with a sample size of 225 cases. There was moderate heterogeneity between studies (*P* = 0.02, *I*^2^ = 71%). The results showed that the pure-tone hearing threshold of the OSAHS group was higher than that of the control group (MD = 9.53; 95% CI 4.34–14.72, Z = 3.6, *P* < 0.001).

There were three studies on the 8,000 Hz hearing test (27, 29, 30) with a sample size of 186 cases. There was no heterogeneity between studies (*P* = 0.52, *I*^2^ = 0%). The results showed that the pure-tone hearing threshold of the OSAHS group was higher than that of the control group (MD = 11.4; 95% CI 7.15–15.65, Z = 5.26, *P* < 0.001).

In conclusion, the pure-tone hearing thresholds at 250, 2,000, 4,000, and 8,000 Hz in OSAHS patients were higher than those of the control group. After subgroup analysis, the heterogeneity of the pure-tone hearing threshold correlation between the OSAHS group and control group was reduced, especially at 4,000 and 8,000 Hz, suggesting that hearing tests of different frequencies may be the source of heterogeneity.

### Sensitivity analysis

**Figure 5:**
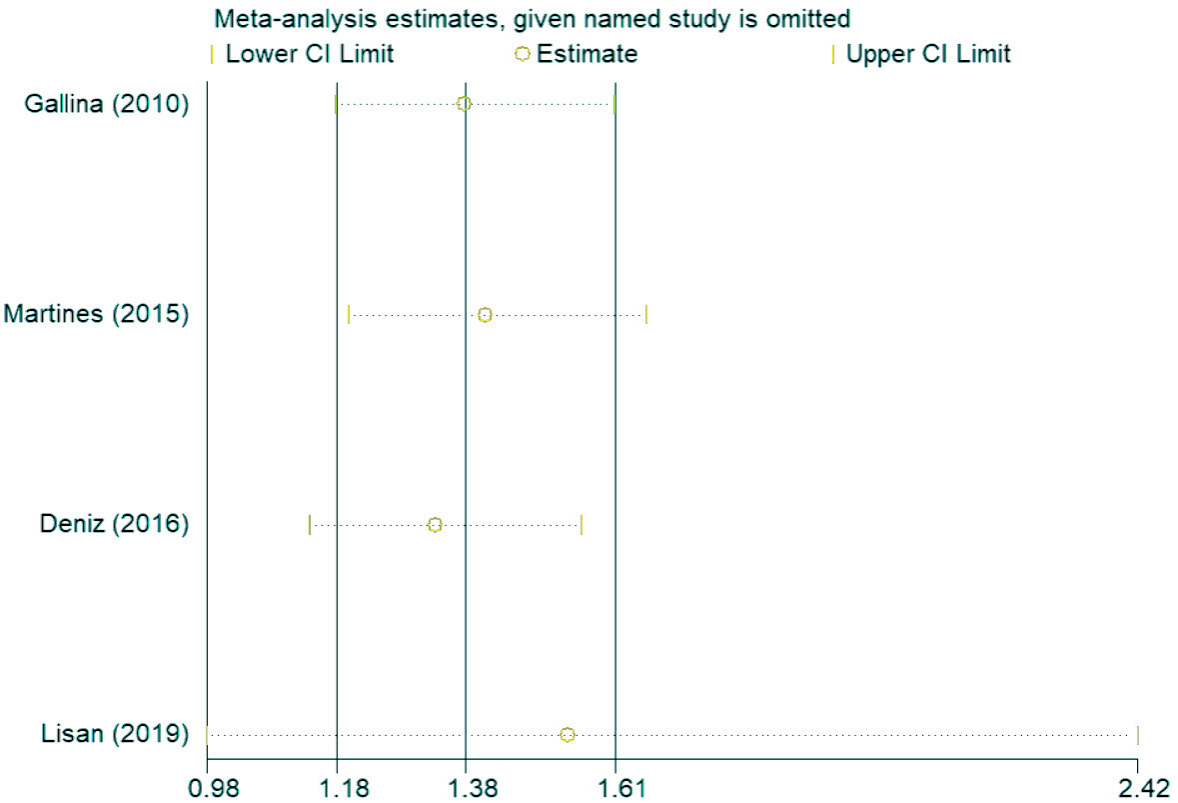
Sensitivity analysis of the incidence of hearing loss in the OASHS group and the control group.

**Figure 6:**
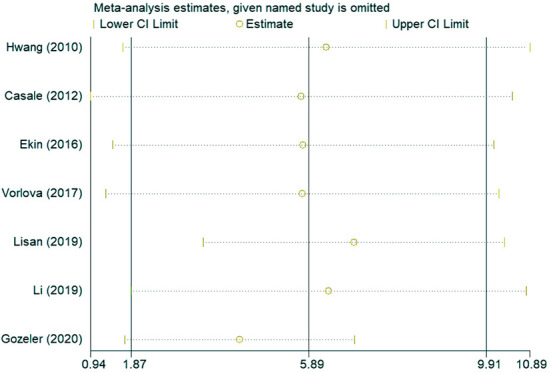
Sensitivity analysis of pure-tone hearing thresholds between OASHS group and control group.

In the sensitivity analysis of the incidence of hearing loss in the OASHS group and control group (Figure 5), the pooled results and cross-study heterogeneity were not significantly changed after excluding any single study. In the sensitivity analysis of the pure-tone hearing threshold in the OASHS group and control group (Figure 6), the combined results and cross-study heterogeneity also did not change significantly after excluding any single study.

### Publication bias

**Figure 7:**
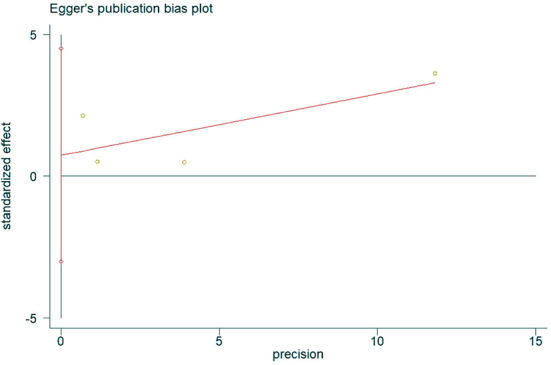
Funnel plot of Egger's method to assess publication bias in incidence of hearing loss in OASHS vs. control.

**Figure 8:**
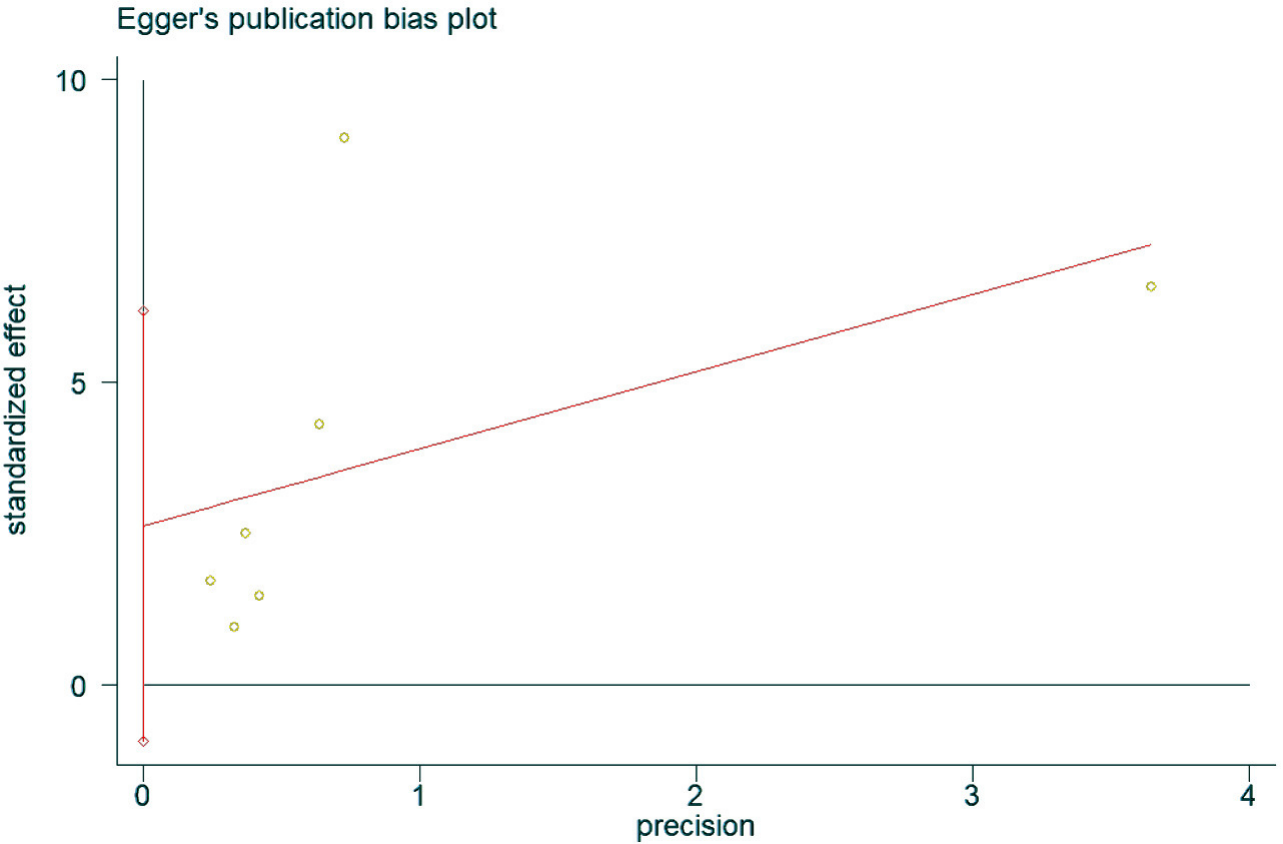
Egger's method for assessing publication bias in OASHS vs. control pure-tone hearing thresholds.

The presence of publication bias was assessed using Egger's method. There were four studies on the incidence of hearing loss in the OASHS group and control group. The result of Egger's method (Figure 7) was *P* = 0.488 > 0.05, indicating no significant publication bias. There were seven studies on the pure-tone hearing threshold between the OASHS group and the control group. The result of Egger's method (Figure 8) was *P* = 0.115 > 0.05, indicating no significant publication bias.

## Discussion

Repeated intermittent hypoxemia and hypercapnia in OSAHS patients can cause pathophysiological changes to many systems in the body (31). Similarly, long-term hypoxia can lead to damage to the cochlear hair cells (32). Due to the physiological characteristics of microcirculation under ischemia, effective collateral circulation cannot be established in a short time, making the system vulnerable to direct and indirect damage from ischemia and hypoxia, and leading to hearing dysfunction (33). At present, many related single-sample studies have been carried out around the world. Due to the limited sample data, the results of each study are quite different. In order to further verify hearing loss in OSAHS patients, this meta-analysis study comprehensively analyzed the research results of literature published in the last 10 years in order to improve clinicians' understanding of hearing loss in OSAHS patients.

This meta-analysis included ten studies, all in English with 7,867 subjects, and we have summarized the effect size value for the mean difference of each study, as shown in Table 3. The methodological quality of the included observational studies was assessed using the NOS scale, which indicated that they were of high quality. The results of the meta-analysis of the incidence of hearing loss in the OSAHS group and control group showed that the incidence was higher in the OSAHS group. The results of the meta-analysis of pure-tone hearing thresholds between the OSAHS group and control group showed that they were higher in the OSAHS group. Subgroup analysis showed that the pure-tone hearing thresholds of 250, 2,000, 4,000, and 8,000 Hz in OSAHS patients were higher than those of the control group, and high-frequency audiometry at 4,000 and 8,000 Hz were significantly increased. Egger's method was used to test whether there was publication bias, and the results confirmed that there was no publication bias in the included literature, indicating that the conclusions of this study are relatively reliable.

**Table 3:** The effect size value for mean differences of the included studies.

**References**	**Outcome**	**Results**
Gallina et al. (21)	Incidence of hearing loss	Incidence of hearing loss: OR = 1.75 (95%CI:0.32-9.67)
Hwang et al. (22)	Average hearing threshold	Average hearing threshold: MD = 3.50(95%CI −1.17-8.17)
Casale et al. (23)	Average hearing threshold	Average hearing threshold: MD = 6.78(95%CI 3.70-9.86)
Martines et al. (24)	Incidence of hearing loss	Incidence of hearing loss: OR: 1.13 (95%CI:0.68-1.87)
Deniz et al. (25)	Incidence of hearing loss	Incidence of hearing loss: OR: 21.63 (95%CI:1.29-363.90)
Ekin et al. (26)	Average hearing threshold	Average hearing threshold: MD = 6.98 (95%CI −1.03-14.99)
Vorlova et al. (27)	Average hearing threshold	Average hearing threshold: MD = 6.80 (95%CI 1.49-12.11)
Lisan et al. (28)	Incidence of hearing loss	Incidence of hearing loss: OR: 1.36 (95%CI:1.15-1.60)
	Average hearing threshold	Average hearing threshold: MD = 1.80 (95%CI 1.26-2.34)
Li et al. (29)	Average hearing threshold	Average hearing threshold: MD = 2.85 (95%CI −3.08-8.78)
Gozeler and Sengoz (30)	Average hearing threshold	Average hearing threshold: MD = 12.45(95%CI 9.75-15.15)

Although there have been recent related studies on OSAHS and hearing loss, the pathophysiological mechanism leading to the high prevalence of hearing loss in OASHS patients has not been determined. There are currently several possible approaches that are considered relevant:

1. Chronic hypoxia damage to the cochlea and vestibular system: Cochlear and vestibular function in OSAHS patients may be affected by intermittent hypoxemia and hypoxia (15, 34). The blood vessels of the inner ear are terminal blood vessels with no collateral circulation between each other. When the inner ear is hypoxic, the cochlea will be seriously affected by hypoxemia, which will further cause different degrees of hearing loss. Fischer et al. (35) found that the blood flow of the right middle cerebral artery was significantly slower than normal during apnea events in sleep, and the hemorheological changes included increased whole blood specific viscosity and increased red blood cell aggregation index. Coupled with the lack of oxygen in the body, this will affect the oxygen and energy supply of the brainstem and cochlea, resulting in a delay in the excitation transmission of the auditory system and a decline in the function of the inner ear. The auditory system is closely related to the vestibular system in embryonic development, innervation, blood supply, anatomical location, etc. Patients with hearing loss are at risk of vestibular dysfunction because damage to the inner ear also extends to vestibular receptors (36). Sensorineural hearing loss (SNHL) is usually secondary to cochlear dysfunction. The anatomical location of the cochlea and vestibular system and the regularity of embryonic development timing offer the possibility that SNHL is associated with vestibular dysfunction (37). Pace et al. (38) used the functional head impulse test (fHIT) to assess vestibular function in patients affected by OSAHS, and the results showed that OSAHS patients may have underlying vestibular dysfunction and can be diagnosed early by fHIT.
2. Neuronal damage caused by chronic hypoxia: Iriz et al. (39) found that the negative impact of OSAHS on the central auditory pathway is the main mechanism of hearing impairment in OSAHS patients. We know that the nerve impulses generated by auditory neurons consume a lot of energy and require a considerable amount of oxygen, so these neurons are more vulnerable to hypoxemia.
3. Snoring noise: Most OSAHS patients experience snoring. The damage to hearing caused by snoring noise is a complex multifactorial mechanism. Noise can damage the microcirculation in the cochlea, which can lead to cochlear ischemia and hypoxia, and cause the degeneration of hair cells and spiral organs (40). In addition, noise can cause serious disorders of the enzymatic system of hair cells and supporting cells, resulting in disturbances to the oxygen and energy metabolism, resulting in cell degeneration and death. The most obvious feature of the hearing curve change caused by noise is the “V”-shaped notch at 4 kHz in the early stages. With the development of the lesion, hearing at 3 kHz and 6 kHz also decreases, and the hearing curve becomes “U”-shaped (40). This is related to the frequency encoding of the basilar membrane of the cochlea progressing from high to low frequencies with distance from the basal end of the cochlea (41), which suggests that chronic snoring noise may cause damage to the high-frequency basal ends of the cochlea, corresponding to the deterioration of extended high-frequency hearing. In addition, Beseky et al. (42) found that different frequencies of sound waves affect different parts of the basilar membrane with the largest amplitude, and high-frequency sound waves cause the largest amplitude of the basilar membrane to be close to the oval window. When the cochlear function is impaired due to hypoxia caused by OSAHS, the first effect is that the high-frequency hearing threshold is significantly improved. The pure-tone audiometry results of this meta-analysis study also showed that the hearing thresholds of the OSAHS patient group at 4 kHz and 8 kHz were the most significantly higher than those of the control group, which is similar to a certain extent, suggesting that snoring may also cause the hearing impairment of OSAHS patients.
4. Neuro-cognitive impairment: According to 70 literatures including the correlation between OSAHS and neurocognitive function, Pollicina et al. (43) found that OSAHS is closely related to cerebrovascular diseases, chronic neurodegenerative diseases and inflammatory diseases, leading to a high risk of cognitive impairment in affected patients. Neuro-cognitive impairment is mostly manifested as auditory dysfunction, such as: decreased speech recognition, decreased or lost the ability to orientate to sounds. A large number of investigations have shown that hearing loss is closely related to cognitive impairment in the elderly. Among the factors that affect the evaluation of cognitive function in the elderly, hearing loss and visual impairment are particularly prominent (44, 45).
5. Upper airway pressure imbalance and Eustachian Tube Dysfunction (ETD): On the one hand, OSAHS episodes are characterized by upper airway pressure imbalance during inspiration. Specifically, this imbalance occurs in the oropharynx and nasopharynx, where the Eustachian tube sits and connects the nasopharynx to the middle ear (46). Opening of the Eustachian tube is caused by elevation of the soft palate and results in ventilation and equilibration of middle ear pressure with ambient pressure to optimize middle ear acoustic transfer function up to the cochlea (47). Air pressure directed toward the nasopharyngeal region has been shown to affect middle ear pressure (48, 49). Therefore, OSAHS and the changes in upper airway pressure it causes during sleep can lead to a negative pressure environment in the middle ear. On the other hand, several OSAHS patients suffer from oro-nasal pathological conditions and other comorbidities, such as nasal congestion, chronic sinusitis, and gastroesophageal reflux (50, 51), which are also thought to be causative genetic factors for ETD (52, 53). Impaired Eustachian tube function is often referred to as Eustachian tube dysfunction. Clinical manifestations of ETD are aural fullness, otalgia and/or hearing loss or could be a “latent” condition (54–56). Magliulo et al. (57) assessed that In patients with obstructive sleep apnea syndrome, several factors (nasal obstruction, increase in upstream airflow resistance, inflammation of the rhinopharynx and the Eustachian tube (ET) orifice mucous) could be responsible for ET collapse, hindering its correct opening to exchange air with the middle ear. This causes impairment of Eustachian tube function and subsequent hearing loss.
6. Metabolic disorders: In recent years, an increasing number of scholars have held that the possible mechanism of OSAHS-induced hearing loss is obesity and abnormal hemorheology associated with OSAHS. This leads to a series of pathophysiological changes, which in turn lead to severe metabolic disorders and multi-system damage in the body. Dhanda and Taheri (58) reported that obesity and lipid metabolism disorders were independent risk factors for hearing impairment. Krajewska et al. (59) found that obese children were more susceptible to diseases such as otitis media, OSAHS and sensorineural hearing loss.

This meta-analysis study explored the correlation of OSAHS with the incidence of hearing loss and pure-tone hearing thresholds, and further analyzed the pure-tone hearing thresholds of different frequencies. In addition, Kayabasi et al. (15) found that the relationship between the severity of OSAHS and hearing impairment may be related to the severity of OSAHS, but related literature is still lacking, some data cannot be extracted and further meta-analysis cannot be carried out. At present, the relationship between the severity of OSAHS and hearing loss has not been clearly elucidated, and more relevant studies are required for further verification in the future.

Limitations of this study: ① this study only included English literature, which may have generated a certain selection bias; ② the sample size of the included studies on the correlation between OSAHS and hearing loss was relatively small, there was a lack of large-scale, multi-center randomized controlled trials, etc.; ③ part of the study population and control population were based on patients who visited a doctor; thus participants were not randomly selected, so there may have been a selection bias; ④ the included studies were case-control studies with weak causality demonstration ability and low overall level of evidence-based medicine; ⑤ Although high-quality literature was included and strict inclusion and exclusion criteria were established, large heterogeneity was still found, and heterogeneity remained after subgroup analysis according to different hearing test frequencies. After analysis, we did not further study the severity of OASHS in the study population, and it may have had a huge impact on the outcome indicators. Due to the limited number of studies, the relevant subgroup analysis could not be carried out, which affected the reliability of the results and may have caused some bias. Therefore, the conclusions herein need to be interpreted with caution, and a large-sample prospective cohort study should be used for further verification in the future.

## Conclusion

In conclusion, the prevalence of hearing loss in OSAHS patients is high, and OSAHS can cause hearing loss. Foreign studies have shown that even with timely symptomatic treatment after the onset of deafness, hearing damage is often irreversible (60). With the continuous improvement of people's living standards and the diversification of various recreational and leisure activities, people are more likely to be affected by different levels of noise, and the prevention and control of hearing loss will become more severe. Therefore, we should face the current situation of the high prevalence of hearing loss in OSAHS patients, carry out interventions for high-risk factors of hearing loss and combine basic public health services to effectively carry out hearing screening and intervention in the population, and actively treat people with hearing loss to effectively prevent its occurrence.

## Data availability statement

The raw data supporting the conclusions of this article will be made available by the authors, without undue reservation.

## Author contributions

CW, FX, MC, CL, XC, XS, and YZ are the guarantor of the manuscript and take responsibility for the content of this manuscript. ZZZ, YW, RC, and CW contributed to the design of the study. QW, HC, JZ, JL, YL, and XO were involved in the data analysis. ZHZ, SL, and RC contributed to the acquisition of primary data. ZZZ, CW, and RC wrote the initial draft of the manuscript. YW, RC, and FX contributed significantly to the revision of the manuscript. All authors read and approved the final manuscript.

## Funding

This study was funded by the Natural Science Foundation of Guangdong Province (2021A1515011373).

## Conflict of interest

The authors declare that the research was conducted in the absence of any commercial or financial relationships that could be construed as a potential conflict of interest.

## Publisher's note

All claims expressed in this article are solely those of the authors and do not necessarily represent those of their affiliated organizations, or those of the publisher, the editors and the reviewers. Any product that may be evaluated in this article, or claim that may be made by its manufacturer, is not guaranteed or endorsed by the publisher.
